# Correction: de Lima et al. Antiviral and Virucidal Activities of *Uncaria tomentosa* (Cat’s Claw) against the Chikungunya Virus. *Viruses* 2024, *16*, 369

**DOI:** 10.3390/v17091244

**Published:** 2025-09-16

**Authors:** Raquel Curtinhas de Lima, Ligia Maria Marino Valente, Débora Familiar Macedo, Luzia Maria de-Oliveira-Pinto, Flavia Barreto dos Santos, José Luiz Mazzei, Antonio Carlos Siani, Priscila Conrado Guerra Nunes, Elzinandes Leal de Azeredo

**Affiliations:** 1 Laboratório das Interações Vírus Hospedeiros, Instituto Oswaldo Cruz, Rio de Janeiro 21040-900, Brazil; raquellima@aluno.fiocruz.br (R.C.d.L.); deborafamiliar@gmail.com (D.F.M.); flaviab@ioc.fiocruz.br (F.B.d.S.);; 2 Instituto de Química, Universidade Federal do Rio de Janeiro, Av. Athos da Silveira Ramos, 149, Rio de Janeiro 21941-909, Brazil; valente@iq.ufrj.br; 3 Laboratório de Tecnologia para Biodiversidade em Saúde, Instituto de Tecnologia de Fármacos, Fundação Oswaldo Cruz, Rio de Janeiro 21041-250, Brazilantonio.siani@fiocruz.br (A.C.S.)

## Text Correction

There was an error in the original publication [1].

In Section 2.10. Flow Cytometry Analysis for CHIKV Antigen Detection, we incorrectly cited BD Biosciences as the manufacturer of the CytoFLEX cytometer. The correct manufacturer is Beckman Coulter, Brea, CA, USA.

A correction has been made to the Materials and Methods section:

**2. Materials and Methods**

*2.10. Flow Cytometry Analysis for CHIKV Antigen Detection*

Cells were analyzed using a CytoFLEX cytometer (Beckman Coulter, Brea, CA, USA).

## Error in Acknowledgments

In the original publication [1], the authors thank the Multi-User Research Facilities of Flow Cytometry and Genomic DNA Sequencing, Oswaldo Cruz Foundation, Rio de Janeiro, Brazil, was not cited. The citation has now been inserted in Acknowledgments, and should read: We thank Thiara Manuele Alves de Souza and Helver Gonçalves Dias for their assistance and technical support. The authors gratefully thank Claire Fernandes Kubelka for their encouragement in natural products’ antiviral action research as well as scientific contributions. The authors thank the Multi-User Research Facilities of Flow Cytometry and Genomic DNA Sequencing, Oswaldo Cruz Foundation, Rio de Janeiro, Brazil.

The authors state that the scientific conclusions are unaffected. This correction was approved by the Academic Editor. The original publication has also been updated.
