# Supplementary material for: Antiviral and Virucidal Activities of Uncaria tomentosa (Cat’s Claw) against the Chikungunya Virus
Source: Viruses. 2024 Feb 27;16(3):369. doi: 10.3390/v16030369 (PMC10974475; doi:10.3390/v16030369)
Supplement: Supplementary file 1 [file viruses-16-00369-s001.zip › viruses-2825530-supplementary.pdf]

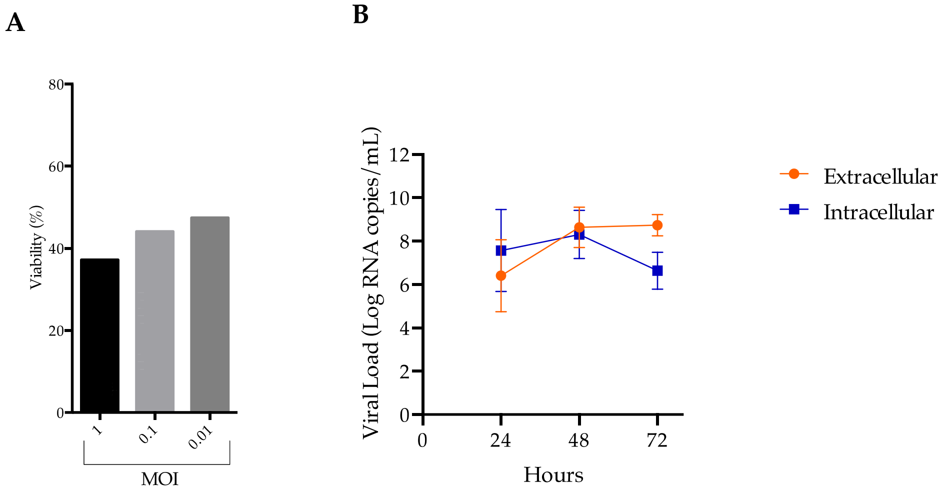

Figure S1. (A) Cell viability in ESCA CHIKV-infected cells after 48h with different multiplicities of infection (MOIs) by MTT assay (B) Quantifying intracellular and extracellular viral CHIKV RNA levels by RT-qPCR after 24,48, and 72h post-infection (p.i). Intracellular and extracellular viral RNA levels were expressed as RNA Log copies per ml (Log RNA copies/mL). The data represent the means (±SD) of at least two independent experiments.

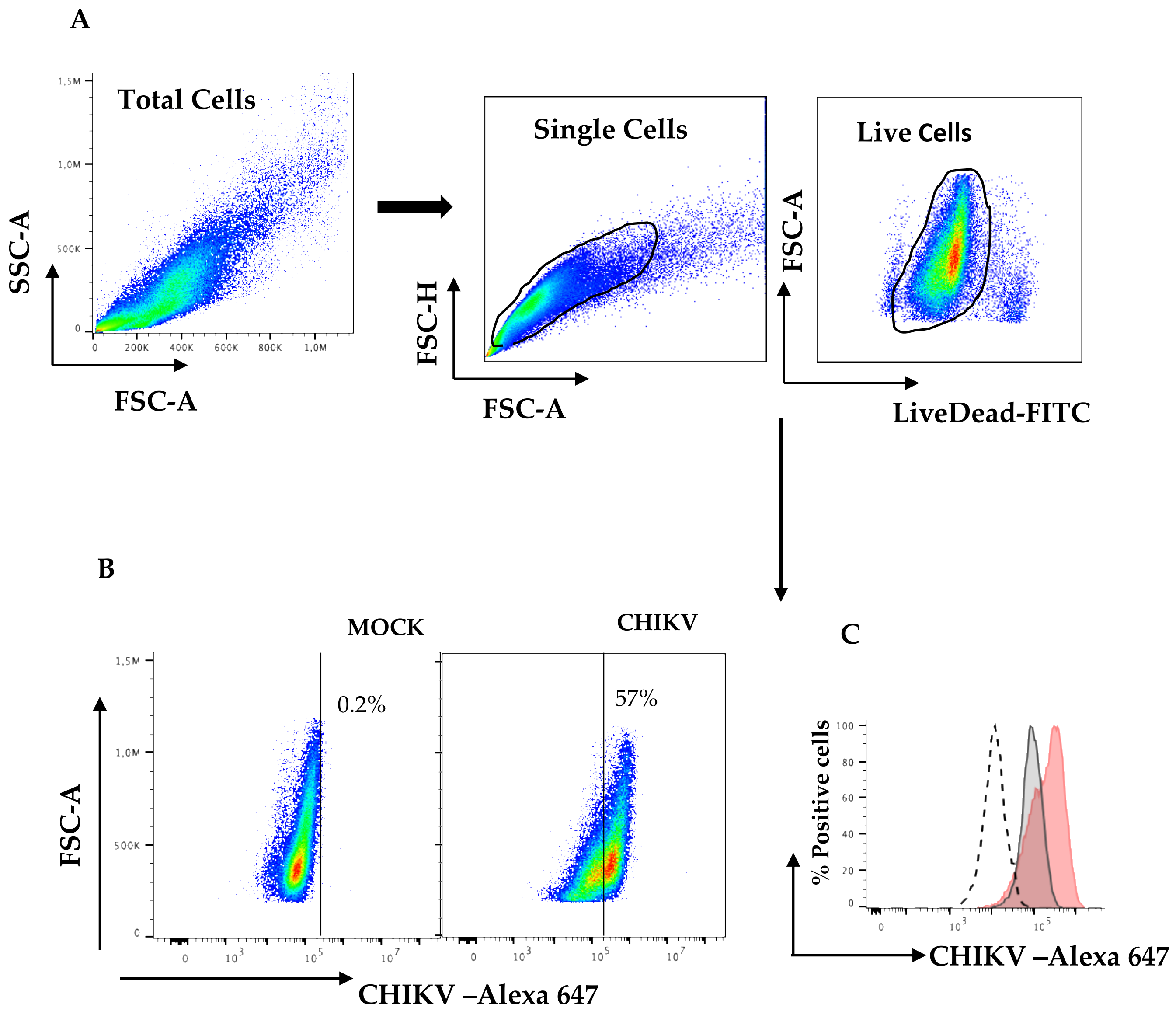

Figure S2: Flow cytometry gating strategy. (A) Total Vero cells were first gated on a forward scatter (FS)/side scatter (SS) plot (a) and then Selection of single cells from a gated area in A, by forward scatter-area vs height and discrimination of live cells from dead cells. (B) Live and positive cells for CHIKV antigen were analyzed FSC /CHIKV from a negative control and positive control. (C) Histograms of CHIKV-positive cells (Vero CHIKV infected, MOI 0.01) versus CHIKV-negative cells (mock). Dashed lines correspond to the isotypic control. Data were analyzed using FlowJo software, version 10.
